# Genetic and environmental influences on height from infancy to early adulthood: An individual-based pooled analysis of 45 twin cohorts

**DOI:** 10.1038/srep28496

**Published:** 2016-06-23

**Authors:** Aline Jelenkovic, Reijo Sund, Yoon-Mi Hur, Yoshie Yokoyama, Jacob v. B. Hjelmborg, Sören Möller, Chika Honda, Patrik K. E. Magnusson, Nancy L. Pedersen, Syuichi Ooki, Sari Aaltonen, Maria A. Stazi, Corrado Fagnani, Cristina D’Ippolito, Duarte L. Freitas, José Antonio Maia, Fuling Ji, Feng Ning, Zengchang Pang, Esther Rebato, Andreas Busjahn, Christian Kandler, Kimberly J. Saudino, Kerry L. Jang, Wendy Cozen, Amie E. Hwang, Thomas M. Mack, Wenjing Gao, Canqing Yu, Liming Li, Robin P. Corley, Brooke M. Huibregtse, Catherine A. Derom, Robert F. Vlietinck, Ruth J. F. Loos, Kauko Heikkilä, Jane Wardle, Clare H. Llewellyn, Abigail Fisher, Tom A. McAdams, Thalia C. Eley, Alice M. Gregory, Mingguang He, Xiaohu Ding, Morten Bjerregaard-Andersen, Henning Beck-Nielsen, Morten Sodemann, Adam D. Tarnoki, David L. Tarnoki, Ariel Knafo-Noam, David Mankuta, Lior Abramson, S. Alexandra Burt, Kelly L. Klump, Judy L. Silberg, Lindon J. Eaves, Hermine H. Maes, Robert F. Krueger, Matt McGue, Shandell Pahlen, Margaret Gatz, David A. Butler, Meike Bartels, Toos C. E. M. van Beijsterveldt, Jeffrey M. Craig, Richard Saffery, Lise Dubois, Michel Boivin, Mara Brendgen, Ginette Dionne, Frank Vitaro, Nicholas G. Martin, Sarah E. Medland, Grant W. Montgomery, Gary E. Swan, Ruth Krasnow, Per Tynelius, Paul Lichtenstein, Claire M. A. Haworth, Robert Plomin, Gombojav Bayasgalan, Danshiitsoodol Narandalai, K. Paige Harden, Elliot M. Tucker-Drob, Timothy Spector, Massimo Mangino, Genevieve Lachance, Laura A. Baker, Catherine Tuvblad, Glen E. Duncan, Dedra Buchwald, Gonneke Willemsen, Axel Skytthe, Kirsten O. Kyvik, Kaare Christensen, Sevgi Y. Öncel, Fazil Aliev, Finn Rasmussen, Jack H. Goldberg, Thorkild I. A. Sørensen, Dorret I. Boomsma, Jaakko Kaprio, Karri Silventoinen

**Affiliations:** 1 Department of Social Research, University of Helsinki, Helsinki, Finland; 2 Department of Genetics, Physical Anthropology and Animal Physiology, University of the Basque Country UPV/EHU, Leioa, Spain; 3 Department of Education, Mokpo National University, Jeonnam, South Korea; 4 Department of Public Health Nursing, Osaka City University, Osaka, Japan; 5 The Danish Twin Registry, Department of Public Health, Epidemiology, Biostatistics & Biodemography, University of Southern Denmark, Odense, Denmark; 6 Osaka University Graduate School of Medicine, Osaka University, Osaka, Japan; 7 Department of Medical Epidemiology and Biostatistics, Karolinska Institutet, Stockholm, Sweden; 8 Department of Health Science, Ishikawa Prefectural Nursing University, Kahoku, Ishikawa, Japan; 9 Department of Public Health, University of Helsinki, Helsinki, Finland; 10 Istituto Superiore di Sanità - National Center for Epidemiology, Surveillance and Health Promotion, Rome, Italy; 11 Department of Physical Education and Sport, University of Madeira, Funchal, Portugal; 12 CIFI2D, Faculty of Sport, Porto, University of Porto, Portugal; 13 Department of Noncommunicable Diseases Prevention, Qingdao Centers for Disease Control and Prevention, Qingdao, China; 14 HealthTwiSt GmbH, Berlin, Germany; 15 Department of Psychology, Bielefeld University, Bielefeld, Germany; 16 Boston University, Department of Psychological and Brain Sciencies, Boston, MA, USA; 17 Department of Psychiatry, University of British Columbia, Vancouver, BC, Canada; 18 Department of Preventive Medicine, Keck School of Medicine of USC, University of Southern California, Los Angeles, California, USA; 19 USC Norris Comprehensive Cancer Center, Los Angeles, California, USA; 20 Department of Epidemiology and Biostatistics, School of Public Health, Peking University, Beijing, China; 21 Institute for Behavioral Genetics, University of Colorado, Boulder, Colorado, USA; 22 Centre of Human Genetics, University Hospitals Leuven, Leuven, Belgium; 23 Department of Obstetrics and Gynaecology, Ghent University Hospitals, Ghent, Belgium; 24 The Charles Bronfman Institute for Personalized Medicine, The Mindich Child Health and Development Institute, Icahn School of Medicine at Mount Sinai, New York, NY, USA; 25 Health Behaviour Research Centre, Department of Epidemiology and Public Health, Institute of Epidemiology and Health Care, University College London, London, UK; 26 King’s College London, MRC Social, Genetic & Developmental Psychiatry Centre, Institute of Psychiatry, Psychology & Neuroscience, London, UK; 27 Department of Psychology, Goldsmiths, University of London, London, UK; 28 State Key Laboratory of Ophthalmology, Zhongshan Ophthalmic Center, Sun Yat-sen University, Guangzhou, China; 29 Centre for Eye Research Australia, University of Melbourne, Melbourne, Australia; 30 Bandim Health Project, INDEPTH Network, Bissau, Guinea-Bissau; 31 Research Center for Vitamins and Vaccines, Statens Serum Institute, Copenhagen, Denmark; 32 Department of Endocrinology, Odense University Hospital, Odense, Denmark; 33 Department of Infectious Diseases, Odense University Hospital, Odense, Denmark; 34 Department of Radiology and Oncotherapy, Semmelweis University, Budapest, Hungary; 35 Hungarian Twin Registry, Budapest, Hungary; 36 The Hebrew University of Jerusalem, Jerusalem, Israel; 37 Hadassah Hospital Obstetrics and Gynecology Department, Hebrew University Medical School, Jerusalem, Israel; 38 Michigan State University, East Lansing, Michigan, USA; 39 Department of Human and Molecular Genetics, Virginia Institute for Psychiatric and Behavioral Genetics, Virginia Commonwealth University, Richmond, Virginia, USA; 40 Department of Human and Molecular Genetics, Psychiatry & Massey Cancer Center, Virginia Commonwealth University, Richmond, Virginia, USA; 41 Department of Psychology, University of Minnesota, Minneapolis, MN, USA; 42 Department of Psychology, University of Southern California, Los Angeles, CA, USA; 43 Institute of Medicine, National Academy of Sciences, Washington, DC, USA; 44 Department of Biological Psychology, VU University Amsterdam, Amsterdam, Netherlands; 45 Murdoch Childrens Research Institute, Royal Children’s Hospital, Parkville, Victoria, Australia; 46 Department of Paediatrics, University of Melbourne, Parkville, Victoria, Australia; 47 School of Epidemiology, Public Health and Preventive Medicine, University of Ottawa, Ottawa, Ontario, Canada; 48 École de psychologie, Université Laval, Québec, Canada; 49 Institute of Genetic, Neurobiological, and Social Foundations of Child Development, Tomsk State University, Russian Federation; 50 Département de psychologie, Université du Québec à Montréal, Montréal, Québec, Canada; 51 École de psychoéducation, Université de Montréal, Montréal, Québec, Canada; 52 Genetic Epidemiology Department, QIMR Berghofer Medical Research Institute, Brisbane, Australia; 53 Molecular Epidemiology Department, QIMR Berghofer Medical Research Institute, Brisbane, Australia; 54 Stanford Prevention Research Center, Department of Medicine, Stanford University School of Medicine, Stanford, CA, USA; 55 Center for Health Sciences, SRI International, Menlo Park, CA, USA; 56 Department of Public Health Sciences, Karolinska Institutet, Stockholm, Sweden; 57 MRC Integrative Epidemiology Unit, University of Bristol, Bristol, U.K; 58 Healthy Twin Association of Mongolia, Ulaanbaatar, Mongolia; 59 Graduate School of Biomedical and Health Sciences, Hiroshima University, Hiroshima, Japan; 60 Department of Psychology, University of Texas at Austin, Austin, TX, USA; 61 Department of Twin Research and Genetic Epidemiology, King’s College, London, UK; 62 School of Law, Psychology and Social Work, Örebro University, Sweden; 63 Washington State Twin Registry, Washington State University - Health Sciences Spokane, Spokane, WA, USA; 64 Washington State Twin Registry, Washington State University, Seattle, WA, USA; 65 Department of Clinical Research, University of Southern Denmark, Odense, Denmark; 66 Odense Patient data Explorative Network (OPEN), Odense University Hospital, Odense, Denmark; 67 Department of Clinical Biochemistry and Pharmacology and Department of Clinical Genetics, Odense University Hospital, Odense, Denmark; 68 Department of Statistics, Faculty of Arts and Sciences, Kırıkkale University, Kırıkkale, Turkey; 69 Departments of Psychiatry, Psychology, and Human and Molecular Genetics, Virginia Institute for Psychiatric and Behavioral Genetics, Virginia Commonwealth University, Richmond, USA; 70 Department of Epidemiology, School of Public Health, University of Washington, Seattle, WA, USA; 71 Novo Nordisk Foundation Centre for Basic Metabolic Research (Section on Metabolic Genetics) and Department of Public Health, Faculty of Health and Medical Sciences, University of Copenhagen, Copenhagen, Denmark; 72 Institute of Preventive Medicine, Bispebjerg and Frederiksberg Hospitals, The Capital Region, Copenhagen, Denmark; 73 National Institute for Health and Welfare, Helsinki, Finland; 74 Institute for Molecular Medicine FIMM, Helsinki, Finland

## Abstract

Height variation is known to be determined by both genetic and environmental factors, but a systematic description of how their influences differ by sex, age and global regions is lacking. We conducted an individual-based pooled analysis of 45 twin cohorts from 20 countries, including 180,520 paired measurements at ages 1–19 years. The proportion of height variation explained by shared environmental factors was greatest in early childhood, but these effects remained present until early adulthood. Accordingly, the relative genetic contribution increased with age and was greatest in adolescence (up to 0.83 in boys and 0.76 in girls). Comparing geographic-cultural regions (Europe, North-America and Australia, and East-Asia), genetic variance was greatest in North-America and Australia and lowest in East-Asia, but the relative proportion of genetic variation was roughly similar across these regions. Our findings provide further insights into height variation during childhood and adolescence in populations representing different ethnicities and exposed to different environments.

Human height is a classic anthropometric quantitative trait for its ease of measurement, approximately normal distribution and relative stability in adulthood, and thus has been the target of extensive research across many fields of science. The study of height has a long standing tradition in genetics; in fact, the field of quantitative genetics was born out of studies of human height in the late 19^th^ and early 20^th^ centuries. Galton^1^ published data as early as 1886 on the relationship between parent and offspring height and inferred that “when dealing with the transmission of stature from parents to children, the average height of the two parents is, … all we need care to know about them”. Later on, Pearson and Lee^2^ presented correlations of height between relatives, also providing evidence for the inheritance of height. In 1918 Fisher^3^ calculated the first heritability estimate of height, i.e. the proportion of total variation explained by genetic variation; in this seminal paper presenting the statistical principles of quantitative genetics, he demonstrated that continuous characters are caused by a combination of many genetic loci with small effects (polygenic inheritance), replacing the blending inheritance hypothesis proposed by Galton. Since then many lines of evidence such as twin, adoption and family studies have estimated the role of genetic factors in the determination of height, showing that it is one of the most heritable human quantitative phenotypes^4^. Interest in the genetic influences on height was renewed when genetic linkage studies enabled research into genetic effects over the whole genome^5^ and genome-wide association (GWA) studies allowed identification of loci consistently associated with height in populations of different ancestry^6^,^7^,^8^,^9^,^10^.

Beside the genetic factors, a multitude of environmental factors can affect height. They can operate during the whole growth period, but infancy is probably the most sensitive phase regarding external influences^11^,^12^. In the presence of adverse environmental conditions, the physical growth of children can decline and even adult height be affected^12^,^13^,^14^. Nutrition and especially lack of dietary protein is universally the most important environmental factor influencing height, but also childhood diseases, in particular infections, can affect growth^11^. These and other proximate biological determinants are further associated with social and economic conditions manifesting as socio-economic differences in height both within and between populations^12^.

Although the heritable nature of height has been recognized for more than one hundred years, only a few studies have explored in detail the genetic variation of height during childhood and adolescence. Twin studies have consistently estimated that the heritability of height is lowest (0.2–0.5) in infancy^15^,^16^,^17^, rapidly increases in childhood with varying values^15^,^16^,^18^, and reaches estimates ranging from 0.70 to 0.90 in adolescence and adulthood^15^,^17^,^19^,^20^,^21^. However, these studies leave unclear whether environmental factors shared by co-twins, which are generally important in infancy and childhood, persist in adolescence or after the cessation of growth^15^,^16^,^17^,^18^,^19^,^20^,^21^,^22^,^23^. A study in four countries with over 12,000 twin pairs from birth to 19 years of age showed that the effect of shared environment remained up through 12 years, and was present again at 16 years^15^. Somewhat different results were observed in a longitudinal study of two Finnish twin cohorts, which found that common environmental factors affected height at different ages in adolescence and early adulthood^20^.

Height is also a classic example of a sexually dimorphic trait; on average, men are taller than women in all human populations^13^. However, much less is known about sex-differences in genetic and environmental contributions to height variation. Greater heritability estimates for males than for females in childhood^15^ and adulthood^21^ have been reported. Also sex-specific genetic effects have been found for height, but the results are inconsistent across studies^18^,^19^,^20^,^21^,^24^.

Further, a greater mean height has been consistently observed in Western populations as compared with East-Asian populations^13^, but most studies on the genetic and environmental factors influencing height variation to date are based on Western populations. A multinational study on adolescent twins from eight countries showed that even when the total variation of height was higher in Western populations, the heritability estimates were largely similar between Western and East-Asian populations^19^. However, these studies did not address the possible differences in the genetic variation pattern between ethnic-cultural groups in childhood and late adolescence/early adulthood.

Using height measures obtained from 45 twin cohorts in 20 countries participating in the COllaborative project of Development of Anthropometrical measures in Twins (CODATwins), we conducted an individual-based analysis of pooled twin cohorts (i) to analyze the genetic and environmental contribution to variation of height from 1 to 19 years of age; (ii) to explore sex-differences in these contributions over each year of age; and (iii) to assess whether this age pattern varies by geographic-cultural region (Europe, North-America and Australia, and East-Asia).

## Results

Descriptive statistics of height by age and sex for the pooled data (all cohorts together) and by geographic-cultural region are presented in Table 1. Mean height expectedly increased with age in both sexes with the exception of the slight decrease observed at 18/19 years of age, which reflects differences in the distribution of different cohorts within each age group. Mean height was greater in boys than in girls; only at the age of 11 and 12 years were girls slightly taller than boys, reflecting the earlier onset of pubertal growth in girls. The difference in mean height between consecutive age groups was very similar in boys and girls during childhood; these mean height differences started to decrease considerably from 12 years in girls and 14 years in boys. The variation of height increased with age and reached the peak at 12 years in girls and 13 in boys, and then decreased slightly. When comparing geographic-cultural regions, mean height was tallest in Europe, somewhat shorter in North-America and Australia and shortest in East-Asia at all ages in boys and girls. The variation of height showed a less clear pattern but was generally greatest in North-America and Australia and lowest in East-Asia.

**Figure 1:**
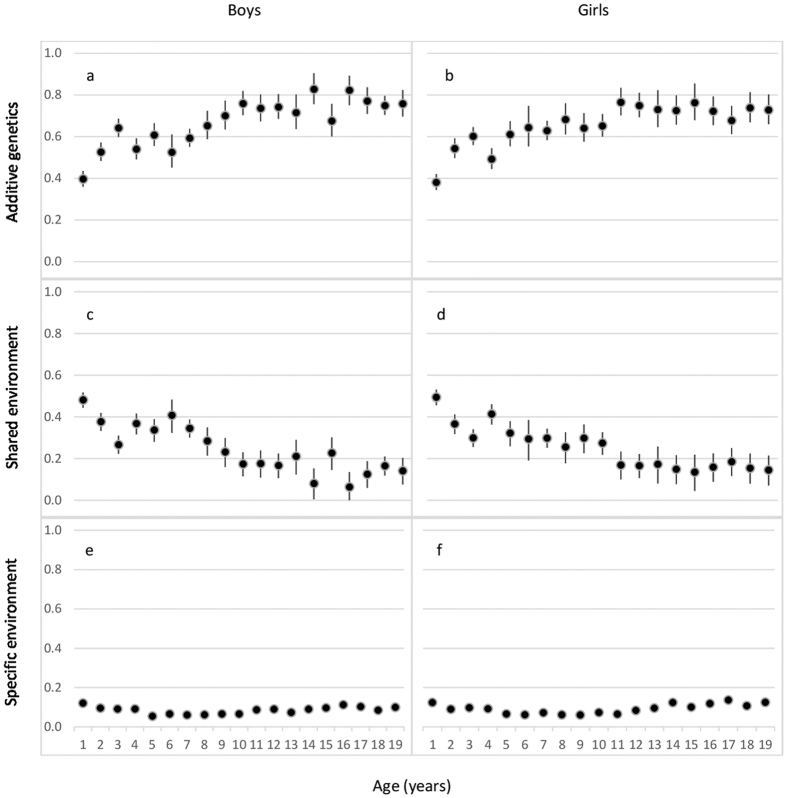
Proportion of height variation explained by additive genetic (**a,b**), shared environmental (**c,d**) and specific environmental (**e,f**) factors with 95% confidence intervals by age and sex for all cohorts together.

**Figure 2:**
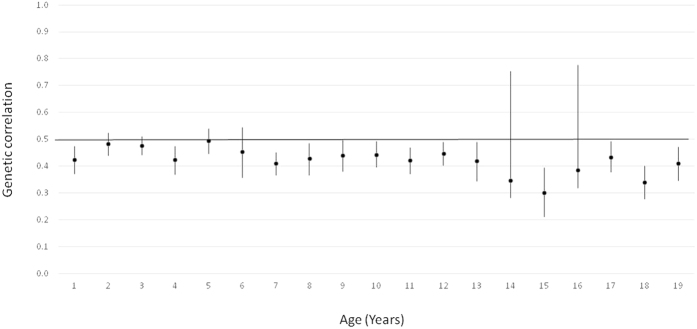
Additive genetic correlations with 95% confidence intervals within opposite-sex DZ pairs by age for all cohorts together.

Figure 1 presents the proportions of height variation explained by additive genetic, common (shared) environmental and unique environmental factors from 1 to 19 years of age in the pooled data (estimates with 95% confidence intervals (CIs) are available in Supplementary Table S1). The proportion of environmental variation shared by co-twins was greatest at age 1 (0.48 in boys and 0.49 in girls), decreased over childhood and stabilized in adolescence, remaining considerable until 19 years (except at ages 14 and 16 in boys) with values generally lower than 0.2. Accordingly, heritability was lowest at age 1 (0.40 in boys and 0. 38 in girls) increased with age in early and middle childhood (~2–5 and 6–8 years of age, respectively) and was generally greater than 0.7 in late childhood (~9–11 years of age) and adolescence; the greatest heritability estimates were found for boys at ages 14 and 16 (0.83 and 0.82, respectively). The proportion of height variation explained by environmental factors unique to each twin individual, which also includes measurement error, did not show any clear age pattern and was largely similar at all ages (0.05–0.14). In spite of the observed sex differences in the relative variance components at most of ages (See Supplementary Table S2), the age pattern was generally similar in boys and girls; the biggest sex-differences were found in late adolescence when the heritability estimates were slightly greater in boys. The point estimates for the genetic correlations within opposite-sex DZ pairs were generally lower than 0.5 suggesting sex-specific genetic effects. There was a trend for these correlations to be lowest in adolescence, although the largest 95% confidence intervals were estimated at 14 and 16 years of age (Fig. 2).

**Figure 3:**
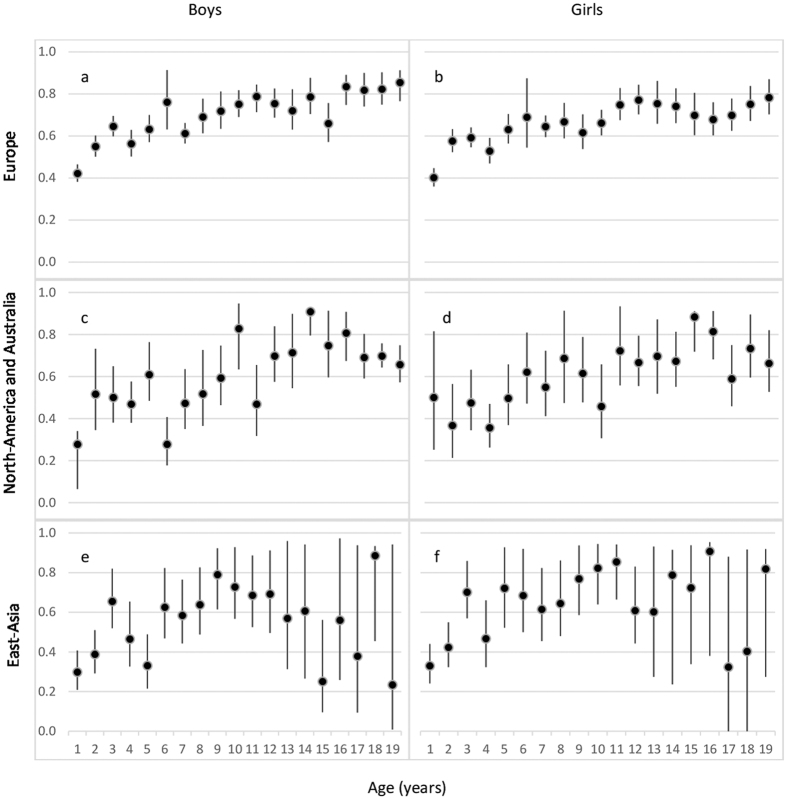
Proportion of height variation with 95% confidence intervals explained by additive genetic factors separately by age and sex in Europe (**a,b**), North America and Australia (**c,d**) and East Asia (**e,f**).

Univariate models for height were then conducted separately in the three geographic-cultural regions. Only the estimates of additive genetic factors are presented in Fig. 3, but all estimates with 95% CIs are available in Supplementary Table S1. The three geographic-cultural regions showed the general trend of increasing proportion of additive genetic factors with age during childhood. Explained by its largest sample size, the pattern in Europe was practically the same to that observed for all cohorts together, but with slightly greater heritability estimates at most ages. In North-America and Australia and East-Asia, heritability estimates in childhood were generally somewhat lower than in Europe. In East-Asia the pattern in adolescence was not so clear because of the smaller sample size leading to wider 95% CIs. In spite of the roughly similar age patterns, the proportions of height variation explained by genetic and environmental factors were different between the geographic-cultural regions (See Supplementary Table S2). The Chinese National Twin Registry was excluded from these analyses because the heritability estimates in that cohort were substantially lower than in other East-Asian cohorts. When data from this cohort was included in the analyses for East-Asia, the proportion of genetic factors decreased and common environmental factors increased considerably; the change in heritability estimates was from 0.1 to 0.3 units depending on the age group (data available on request).

**Figure 4:**
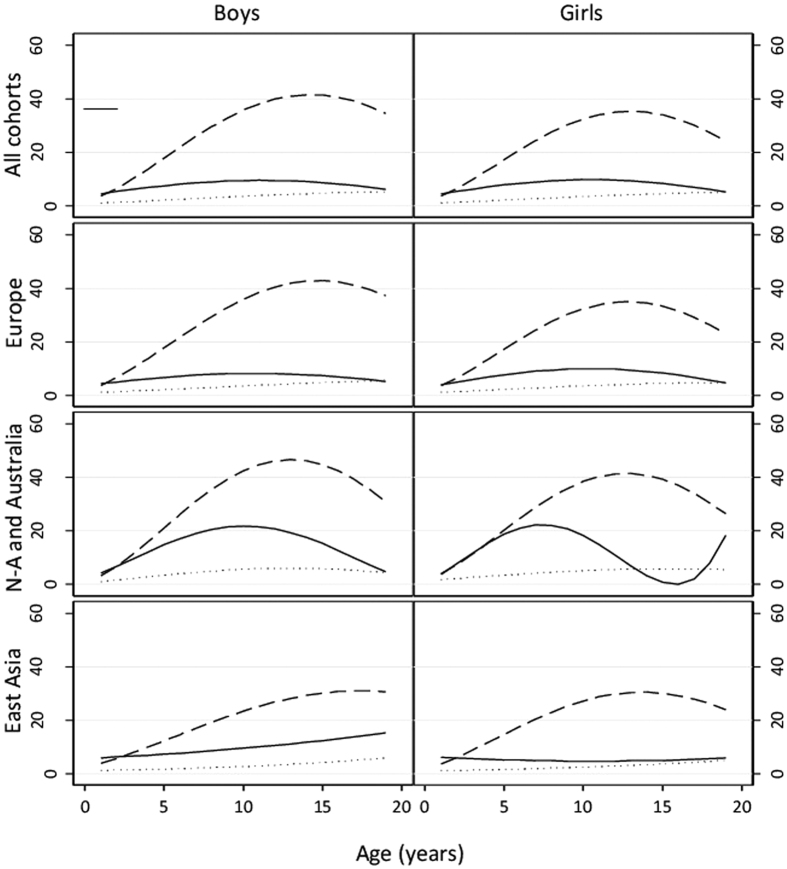
Change of additive genetic (dash line), common environmental (solid line) and unique environmental (dot line) variance with increasing age in quadratic gene-environment interaction model in Europe, North America and Australia and East Asia.

Finally, we studied how age modifies the genetic and environmental variances of height by using gene-age interaction analysis, with data pooled across all age groups. Figure 4 shows the change in the predicted raw genetic and environmental variances in height as a function of age (parameter estimates with 95% CIs are available in Supplementary Table S3). Additive genetic variation increased steadily from age 1, reached its peak at 14 years in boys and 13 years in girls and then decreased again in the pooled data; however, common and unique environmental variation were largely similar across ages. When stratified by geographic-cultural region, genetic variation was largest in North-America and Australia, somewhat lower in Europe, and lowest in East-Asia, particularly for boys. The pattern of genetic variance increasing to a maximum and thereafter decreasing was consistent across the regions. Also common environmental variation was greatest in North-America and Australia, reaching the peak at 10 years in boys and 7 years in girls, whereas in Europe and East-Asia it was similar across ages. Unique environmental variation showed a similar pattern and magnitude in the three geographic-cultural regions. The differences between the regions were highly statistically significant in boys [difference in −2 log-likelihood values (Δ − 2LL) = 1257, difference in degrees of freedom (Δd.f.) = 30, p-value < 0.0001)] and girls (Δ − 2LL = 1364, Δd.f. = 30, p-value < 0.0001). When comparing sexes, in Europe and North-America and Australia there was a trend toward a greater genetic variation for boys than for girls, which increased with age. In East-Asia, however, genetic variation was slightly greater for girls until 14 years of age and for boys in late adolescence.

## Discussion

The present study of 180,520 paired measurements from 86,037 complete twin pairs in 20 countries revealed that environmental factors shared by co-twins contribute to the inter-individual variation in height from infancy to early adulthood. The relative proportion of common environmental factors was greatest during the first years of life, representing almost half of the variation at age 1, and decreased over childhood and adolescence. The interpretation of these results, however, deserves some caution. It has been questioned whether twin studies are suitable for estimating heritability of height in infancy, since early growth patterns in twins differ considerably from singleton growth patterns^25^. Prenatal environmental factors can act very differently on MZ twins leading to differences in body size within pairs (the most extreme case is the twin-to-twin transfusion syndrome). This is an important issue because in the classical twin design heritability is estimated by comparing the resemblance of MZ and DZ twin pairs, and thus body size differences in MZ pairs will result in lower heritability estimates. Since children may take several years to fully catch-up after birth, the high proportion of height variation explained by the shared environment in infancy may still reflect these prenatal environmental factors. Among other possible explanations, it might be that the shared environment represents the effects of gestational age or the effects of the higher measurement error (correlated in twins) at earlier ages.

The influence of the shared environment on height variation up to 19 years, which is consistent with previous studies in adolescents^19^ and adults^21^ with enough statistical power to detect this component, suggests that adult height variation reflects childhood living conditions. Studies have shown that the secular trend in adult height occurs during the first two years of life mainly due to increases in leg length^26^. A plausible explanation is that the period of most rapid growth, when the effect of an adverse environment is strongest, coincides with the period when most growth takes place in the long bones of the legs^26^. Multinational studies analyzing the genetic and environmental influences on body length segments, particularly leg length, are thus needed to disentangle the aetiology of total height variation. The small but considerable effect of unique environment on height variation, very similar across ages, may partly be due to measurement error, which is modelled as part of unique environmental factors. However, it is likely that it also reflects real environmental factors, for example, different exposure to childhood diseases.

A recent and large meta-analysis of twin correlations and variance components for 17,804 traits carried out separately in four age groups (0–11, 12–17, 18–64 and 65+ years) showed that the heritability estimate of height at 12–17 years was considerably greater than at 0–11 years^27^. Given the rapid growth that occurs in infancy, childhood and adolescence, in this individual-based pooled analysis we analyzed the heritability of height in one year age groups. We found that genetic contributions increase over childhood with heritability estimates in the range of previous studies in children and adults^15^,^16^,^18^,^20^,^21^. GWA studies have identified many common genetic variants for adult height. The most recent GWA meta-analysis in 253,288 individuals of European ancestry identified 697 genome-wide significant SNPs in 423 loci that together explained one-fifth of the heritability for adult height^10^. Further, in a study using whole-genome sequencing data from 44,126 unrelated individuals, all imputed variants explained 56% of variance for height suggesting that missing heritability is negligible for human height^28^. However, much less is known on the genetics of height in children. Van der Valk *et al*.^29^ found that polygenic scores based on 180 SNPs previously associated with adult height explained 2.95% of the variance of infant length, and that of 180 known adult height loci, only 11 were genome-wide significantly associated with infant length.

The pattern of total height variation across ages was largely driven by genetic variance. The most consistent result is the increasing genetic variance with age, reaching its peak at around 13 years in girls and 14 years in boys. After that point, even if mean height continued to increase, genetic variance started to decrease in such a way that in late adolescence the magnitude was similar to that before pubertal events start. Adolescence is characterized by the onset of puberty and the occurrence of growth spurts. Although a secular and population-dependent decline has been observed in the age at onset of pubertal growth spurt and peak height velocity since the mid 1900s^30^,^31^, the pubertal height spurt generally begins at age 10–11 years in girls and 11–13 years in boys and reaches peak height velocity at about 12 years and 14 years, respectively^13^,^30^. In this study, twins within age groups are at various stages of puberty. In addition to the substantial heritability reported for pubertal timing^32^, a genome-wide genetic correlation (0.13) between age at menarche and adult height has also been found^33^. In fact, a genome-wide association meta-analysis showed that five loci associated with pubertal timing impacted multiple aspects of growth, both before and during puberty^34^. Therefore, it is possible that some of the genetic variance in height at these ages is confounded with genetic variance in pubertal events.

In spite of the largely similar age patterns observed in boys and girls, boys showed somewhat greater heritability estimates and genetic variation, especially in late adolescence. Greater heritability estimates in boys than in girls have previously been reported from birth through 19 years^15^ and in adulthood^21^. Moreover, some studies have shown a sex-specific genetic effect on height variation in adolescents^19^ and adults^24^. It is clear that both of the sex chromosomes are implicated in determining mean height. Short stature has been demonstrated in females with Turner syndrome who have only one X chromosome^35^ and taller stature seen in XYY men compared with XY men^36^. However, sex chromosomes have also been associated with height variation; for example, Gudbjartsson *et al*.^37^ identified 27 regions of the genome including a locus on X chromosome that together explained around 3.7% of the population variation in height. In our multinational data, the lowest genetic correlations within opposite-sex DZ pairs were found at 14–16 years of age and again at 18 years, suggesting that sex-specific genes have a role in the genetic variation of height not only during puberty, but also in late adolescence.

Comparison between geographic-cultural regions showed that mean height was greatest in Europe, somewhat shorter in North-America and Australia and shortest in East-Asia, but total variance was largest in North-America and Australia. Accordingly, genetic variation was also greatest in North-America and Australia and lowest in East-Asia. However, the relative proportions of additive and environmental variations were more similar in the different geographic-cultural regions. These results are consistent with a previous comparative twin study which found that the mean and variance of height were larger in Caucasian than in East-Asian populations in adolescence, but the heritability estimates were still at the same level^19^. An important proportion of the differences in total variances between geographic-cultural regions were attributable to genetic differences. It may be that allelic frequencies and effects of the genes involved in height vary between Europeans, North-Americans and Australians and East-Asians, leading to differences in genetic variation between the three population groups. A recent study across 14 European countries found that many independent loci contribute to population genetic differences in height, and estimated that these differences account for 24% of the captured additive genetic variance^38^. However, a major part of the differences in genetic variation may also be because of gene-environment interactions modelled as part of the additive genetic component in our model. That is, the higher genetic variation observed in Caucasians could arise because there is a set of genes expressed more strongly in Western environments. For example, a study of adults of Japanese descent living in the United States and native Japanese found that Japanese men and women were shorter than Japanese-Americans, suggesting that environmental factors play a role in physical growth^39^. Analyzing this question in detail would require collection of twins or GWA studies in unrelated individuals with East-Asian origin living in a Western environment.

The study in Caucasian and East-Asian populations showed that approximately 91% of the differences in the total variance between these two population groups was attributable to genetic variances^19^. However, our study found that shared environmental variance also differed between geographic-cultural regions. The lower shared environmental variance observed in East Asian girls and greater in North-America and Australia during childhood may reflect cultural differences in terms of nutrition and other environmental resources. It is also important to note that we limited our East-Asian cohorts to affluent East-Asian populations including the Shandong and Guangdong provinces but excluding poorer areas of China. As reported previously, the heritability estimates of height were considerably lower and common environmental estimates higher in the poorer areas^40^, which may indicate larger differences between families in nutrition and infection history in these areas of China. This emphasizes the need to collect data on twins living under different environmental exposures.

The main strength of the present study is the very large sample size of our multinational database of twin cohorts, with height data from 1 through 19 years of age, allowing a more detailed investigation of the genetic and environmental contributions to individual differences in height during childhood and adolescence than in the previous studies. Twin participants are from 20 different countries, thereby making it possible to stratify the analyses by regions representing different ethnicities and environments. Important advantages of individual-based data are better opportunities for statistical modelling and lack of publication bias. However, our study also has limitations. The equal-environment assumption, upon which twin methodology is based, assumes that MZ and DZ twins are equally exposed to environmental factors relevant to the outcome. If equal-environment assumption is violated, it should be seen as differences in variances between MZ and DZ twins, but we did not find such evidence. In the classical twin design phenotypic assortment increases DZ correlations and thus inflates the common environmental component when not accounted for in the modelling. Assortative mating is well recognized for height, and when the potential underestimation of heritability estimates was corrected using a sample of twins and their parents^41^, these authors showed that doing so increased the heritability estimates from 0.75 to 0.85. In our database we do not have information on parental height and thus could not take into account assortative mating, which may thus explain part of the shared environmental variation. A recent study showed that increased homozygosity, which is influenced by inbreeding, was associated with decreased height and that the effect sizes were similar across different continental groups and populations with different degrees of genome-wide homozygosity^42^. These authors thus suggested that homozygosity, rather than confounding as a result of environmental or additive genetic effects, directly contributes to phenotypic variance^42^. Further, most of the height measures were self-reported^43^, which are prone to error and can bias our analyses toward lower heritability estimates and higher estimates of unique environmental effects. Finally, countries and/or ethnic-cultural regions are not equally represented, and the database is heavily weighted towards populations following the Westernized lifestyle; even when the large majority of the twin cohorts in the world participated in this project, our data still had limited power for East-Asia especially in adolescence. An even bigger problem is that there are few data available from South-Asia, Middle-East and Africa and no data from South-America. This demonstrates the need for new data collections in these regions.

Our findings provide further insights into height variation during childhood and adolescence in populations representing different ethnicities and exposed to different environments. Worthwhile objectives for future research are to study whether the same genetic and environmental factors contributing to height variation operate throughout time or new genes or new environmental factors start to operate at different ages, and to analyze the heritability of growth in height. Further, a major challenge in future studies with more information on birth and pregnancy related variables is to explore the reasons for the low heritability of height at young ages.

In conclusion, environmental factors shared by co-twins exert their strongest influence on height variation in childhood, but these effects remain until the onset of adulthood. Genetic variation in height increased steadily during childhood and reached its peak at around 13 years in girls and 14 years in boys, which may be confounded with genetic variation in pubertal events. Especially in adolescence, there was a trend toward somewhat greater genetic variation in boys than in girls, and part of the genetic variation of height was sex-specific. Genetic variation of height was larger in North-America and Australia and Europe compared with East-Asia, but the relative proportions of genetic and environmental variations between these three geographic-cultural regions were roughly similar. These findings suggest that, in spite of different ethnicities and environmental exposures, genetic factors play a major role on height variation in adolescence and early adulthood, but environmental factors shared by co-twins are also important.

## Methods

### Ethics

All participants were volunteers and gave their informed consent when participating in the study. No experimental data were asked and thus we did not ask ethical approval. Only a limited set of observational variables and anonymized data were delivered to the data management centre at University of Helsinki. The pooled analysis was approved by the ethical committee of Department of Public Health, University of Helsinki, and the methods were carried out in accordance with the approved guidelines.

### Sample

This study is based on the data from the CODATwins project described elsewhere^43^. Briefly, the CODATwins project was intended to recruit all twin projects in the world with information on height and weight measurements. For the present analyses, we selected height measurements at ages from 0.5 to 19.5 years (n = 420,707). Age was classified to single-year age groups (e.g., age 1 refers to 0.5–1.5 years range). Impossible values and outliers were checked by visual inspection of histograms for each age and sex group and were removed to obtain an approximately normal distribution (0.3% of the measurements). Since individuals in longitudinal studies have more than one measurement over time, analyses were restricted to one observation per individual in each age group. Analyses were additionally restricted to having at least 50 measurements per cohort. Finally we had data from 45 cohorts in 20 countries: one cohort from Africa (Guinea Bissau Twin Study), two cohorts from Australia (Peri/Postnatal Epigenetic Twins Study, and Queensland Twin Register), seven cohorts from East-Asia (Chinese National Twin Registry, Guangzhou Twin Eye Study, Japanese Twin Cohort, Mongolian Twin Registry, Qingdao Twin Registry of Children, South Korea Twin Registry, and West Japan Twins and Higher Order Multiple Births Registry), 20 cohorts from Europe (Adult Netherlands Twin Registry, Berlin Twin Register, Bielefeld Longitudinal Study of Adult Twins, Danish Twin Cohort, East Flanders Prospective Twin Survey, Finnish Older Twin Cohort, FinnTwin12, FinnTwin16, Gemini Study, Genesis 12–19 Study, Hungarian Twin Registry, Italian Twin Registry, Portugal Twin Cohort, Swedish Twin Cohorts, Swedish Young Male Twins Study of Adults, Swedish Young Male Twins Study of Children, TCHAD-study, Twins Early Developmental Study, TwinsUK, and Young Netherlands Twin Registry), two cohorts from Middle-East (Longitudinal Israeli Study of Twins and Turkish Twin Study) and 13 cohorts from North-America (Boston University Twin Project, California Twin Program, Colorado Twin Registry, Michigan Twins Study, Mid Atlantic Twin Registry, Minnesota Twin Family Study, NAS-NRC Twin Registry, Quebec Newborn Twin Study, SRI-international, Texas Twin Project, University of British Columbia Twin Project, University of Southern California Twin Study, and University of Washington Twin Registry). A more detailed description of the participating twin cohorts was presented previously^43^.

During the course of the study, we found that the heritability estimates of height were substantially lower in the Chinese National Twin Registry than in other East-Asian cohorts, as also reported previously^40^. Because of this heterogeneity, we did not include the data from the Chinese National Twin Registry in the reported analyses but tested how it would change the results in East-Asia. The final database comprised information on 86,037 different complete twin pairs with a total of 180,520 paired measurements (39% monozygotic (MZ), 34% same- sex dizygotic (SSDZ) and 27% opposite-sex dizygotic (OSDZ) twin pairs); that is, since some twin pairs have measurements at different ages, our database is based on measurement pairs. In order to analyze possible ethnic-cultural differences in the genetic and environmental contribution on height, cohorts were pooled in three groups according to their geographical and cultural characteristics: Europe (20 cohorts), North-America and Australia (15 cohorts) and East-Asia (6 cohorts) with 131,856, 29,856 and 17,924 paired measurements, respectively. In the additional analyses including the Chinese National Twin Registry, the number of pairs in East-Asia was 27,067. The cohort from Africa and the two from Middle-East were not included in these sub-analyses by geographic-cultural region because the data is too small to study these two areas separately.

### Statistical analyses

To analyze genetic and environmental influences on the variation of height, we used classic twin modelling based on linear structural equations^44^. Briefly, the analysis is based on the fact that MZ twins share the same gene sequence, whereas DZ twins share, on average, 50% of their genes identical-by-descent. On this basis, it is possible to divide the total variation of height into variance due to additive genetic effects (A: correlated 1.0 for MZ and 0.5 for DZ pairs), dominance genetic effects (D: 1.0 for MZ and 0.25 for DZ pairs), common (shared) environmental effects (C: by definition, correlated 1.0 for MZ and DZ pairs) and unique (non-shared) environmental effects (E: by definition, uncorrelated in MZ and DZ pairs). However, since our data included only twins reared together, we cannot simultaneously estimate common environmental and dominance genetic effects. All genetic models were fitted by the OpenMx package (version 2.0.1) in the R statistical platform^45^ using the maximum likelihood method.

Prior to conducting the modelling, height values were adjusted using linear regressions and the resulting residuals were used as input phenotypes for the following analyses. Adjustment was carried out for birth year, exact age at the time of the measurement and study cohort within one-year and sex groups in univariate analyses, and for birth year and cohort in gene-environment interaction analyses.

The ACE sex-limitation model was selected as a starting point of the univariate modelling based on the following criteria: (i) MZ within-pair correlations were clearly higher than DZ correlations consistent with the influence of genetic effects, (ii) the magnitude of the difference between MZ and DZ correlations (rDZ > 1/2 rMZ) indicated the presence of common environmental effects and (iii) the lower within-pair correlations for OSDZ than for SSDZ twins observed at most ages suggested the presence of sex-specific genetic effects (See Supplementary Table S4). Previous findings from this international database showed that both male and female DZ twins have greater height than MZ twins in these age groups^46^, and thus different means for MZ and DZ twins were allowed. The fit of the univariate models for height at each one-year age group is shown in Supplementary Table S2. In the present study, the equal-environment assumption was tested by comparing the ACE model to the saturated model. The fit of the model after Bonferroni correction of multiple testing did not worsen at most ages, which suggested that the assumption of equality of variances between MZ and DZ twins was not violated. When fixing A, C and E parameters to be the same in boys and girls, the fit of the model was poorer at many ages, particularly from the beginning of adolescence, suggesting that these variance components differ between sexes. We additionally fitted a scale model allowing for different sizes of variance components but fixing the relative size of these components to be equal. Since this model also showed differences, we decided to present the results separately for boys and girls. Sex-specific genetic effects were considerable at some ages, and thus all modelling results are presented in sex-limited form for consistency. Finally, comparative model fitting revealed that the C parameter could be not excluded from the model without a significant (P < 0.001) deterioration in fit for all ages.

In order to study how age modifies the genetic and environmental variances of height, we additionally conducted gene–environment interaction modelling using age as an environmental modification factor^47^. The advantage of the gene-environment interaction model, as compared to fitting a series of univariate models, is that it estimates a fewer number of parameters and thus has more statistical power to analyze the age patterns in genetic and environmental variances. The parameter estimates differed in boys and girls (Δ − 2LL = 407, Δd.f. = 15, p-value < 0.0001) and thus sex-specific models were conducted. Because the size of the sex-specific genetic effect varied according to age, only same-sex pairs were included in these age-moderation analyses. In addition to linear effects of age, quadratic age effects were also included on the variance components since they were highly statistically significant in boys (Δ − 2LL = 3298, Δd.f. = 3, p-value < 0.0001) and girls (Δ − 2LL = 4091, Δd.f. = 3, p-value < 0.0001).

## Additional Information

**How to cite this article**: Jelenkovic, A. *et al*. Genetic and environmental influences on height from infancy to early adulthood: An individual-based pooled analysis of 45 twin cohorts. *Sci. Rep.* **6**, 28496; doi: 10.1038/srep28496 (2016).

## Supplementary Material

Supplementary Information

## Figures and Tables

**Table 1:** Descriptive statistics of height by age, sex and geographic-cultural region.

	All cohorts			Europe			North-America and Australia			East-Asia		
	N	mean	SD	N	mean	SD	N	mean	SD	N	mean	SD
Boys												
Age 1	15512	74.3	4.3	12674	75.3	3.3	547	71.6	8.8	2213	69.4	3.7
Age 2	12667	87.0	4.3	10357	87.7	3.9	607	85.2	4.7	1629	83.4	4.6
Age 3	16840	96.3	4.4	13761	96.8	4.3	1050	96.3	5.1	1987	93.4	3.8
Age 4	9719	102.4	5.3	7244	103.0	4.9	1481	101.4	6.4	986	99.6	4.6
Age 5	7987	111.5	6.0	6014	112.6	5.5	1080	108.9	6.5	870	106.5	4.6
Age 6	3167	115.5	6.7	1328	118.3	5.6	797	114.4	8.4	986	112.6	4.9
Age 7	13396	124.3	6.6	11136	125.3	6.1	707	121.5	8.8	1240	118.1	5.2
Age 8	6298	128.8	6.5	4333	130.3	5.8	679	129.6	7.2	1243	123.8	5.2
Age 9	6870	134.2	7.0	4212	135.8	6.6	1398	133.9	7.4	1227	129.4	5.5
Age 10	11228	140.9	7.2	8902	142.1	6.8	839	139.2	7.4	1358	135.2	6.1
Age 11	8839	144.6	7.4	6470	145.6	7.1	951	144.4	8.4	1415	140.5	6.4
Age 12	11599	151.9	8.1	8342	152.7	7.9	2197	151.8	8.2	1060	146.5	7.1
Age 13	4745	158.9	9.3	3322	159.7	8.8	1124	157.6	10.1	299	154.7	9.6
Age 14	8896	165.9	8.9	6308	166.0	9.1	2422	165.7	8.4	166	164.2	7.2
Age 15	5307	171.8	8.7	3782	172.3	8.8	1377	171.3	8.4	137	166.7	7.3
Age 16	8013	175.7	7.5	5806	176.2	7.4	2070	174.9	7.6	120	168.4	6.1
Age 17	9829	177.0	7.4	6795	178.2	7.1	2918	174.4	7.5	100	171.5	6.8
Age 18	14722	176.1	7.6	6514	179.6	6.9	8063	173.4	7.1	118	170.7	5.6
Age 19	8873	177.7	7.8	4822	180.0	6.9	3923	174.9	7.8	117	174.4	5.3
Girls												
Age 1	15520	72.9	4.3	12498	74.1	3.0	583	70.3	9.0	2355	67.8	3.9
Age 2	12223	85.9	4.3	9885	86.7	3.8	579	84.1	4.8	1681	81.9	4.4
Age 3	17216	95.3	4.5	13949	95.8	4.4	1086	95.1	5.5	2137	92.3	3.6
Age 4	9571	101.3	5.2	7144	101.8	4.9	1411	100.1	6.3	1000	98.6	4.5
Age 5	7817	110.6	6.2	5884	111.9	5.6	1010	107.5	7.0	902	105.5	4.6
Age 6	2807	114.6	6.5	886	117.9	5.3	785	113.9	8.0	1070	112.3	4.8
Age 7	13736	123.5	6.6	11368	124.5	6.2	719	120.4	7.5	1358	117.5	5.0
Age 8	6072	127.8	6.7	3979	129.5	5.9	681	127.6	8.0	1389	123.2	5.4
Age 9	6642	133.3	7.1	3868	134.8	6.8	1362	133.3	7.5	1375	129.0	5.6
Age 10	11298	140.5	7.4	8882	141.6	7.0	817	138.7	8.3	1468	135.4	6.5
Age 11	8743	145.1	7.7	6288	145.8	7.6	953	145.2	8.5	1499	142.1	7.0
Age 12	11873	152.9	8.2	8376	153.7	8.2	2327	152.9	7.9	1170	147.7	6.8
Age 13	4599	158.3	7.6	3078	159.5	7.4	1216	156.4	7.6	305	153.5	6.3
Age 14	9388	162.2	6.7	6644	162.6	6.8	2578	161.4	6.5	166	157.5	6.0
Age 15	5299	164.5	6.9	3736	165.5	6.7	1379	162.6	6.7	173	157.6	5.6
Age 16	8801	164.9	6.5	6516	165.4	6.2	2128	163.9	6.8	150	158.4	6.0
Age 17	9203	165.8	6.5	7523	166.1	6.3	1534	164.6	7.0	122	159.3	5.5
Age 18	7704	166.3	6.7	5504	167.1	6.5	2037	164.6	7.0	130	159.5	4.8
Age 19	8021	166.2	6.7	5582	167.2	6.4	2297	164.4	6.9	127	158.4	5.3

N: number of twin individuals; SD: standard deviation.
