# Supplementary material for: Genetic and environmental influences on height from infancy to early adulthood: An individual-based pooled analysis of 45 twin cohorts
Source: Sci Rep. 2016 Jun 23;6:28496. doi: 10.1038/srep28496 (PMC4917845; doi:10.1038/srep28496)
Supplement: Supplementary Information [file srep28496-s1.pdf]

Genetic and environmental influences on height from infancy to early adulthood: An individual-based pooled analysis of 45 twin cohorts

Aline Jelenkovic* (1) (2), Reijo Sund (1), Yoon-Mi Hur (3), Yoshie Yokoyama (4), Jacob v. B. Hjelmborg (5), Sören Möller (5), Chika Honda (6), Patrik KE Magnusson (7), Nancy L Pedersen (7), Syuichi Ooki (8), Sari Aaltonen (1) (9), Maria A Stazi (10), Corrado Fagnani (10), Cristina D'Ippolito (10), Duarte L Freitas (11), José Antonio Maia (12), Fuling Ji (13), Feng Ning (13), Zengchang Pang (13), Esther Rebato (2), Andreas Busjahn (14), Christian Kandler (15), Kimberly J Saudino (16), Kerry L Jang (17), Wendy Cozen (18) (19), Amie E Hwang (18), Thomas M Mack (18) (19), Wenjing Gao (20), Canqing Yu (20), Liming Li (20), Robin P Corley (21), Brooke M Huibregtse (21), Catherine A Derom (22) (23), Robert F Vlietinck (22), Ruth JF Loos (24), Kauko Heikkilä (9), Jane Wardle † (25), Clare H Llewellyn (25), Abigail Fisher (25), Tom A McAdams (26), Thalia C Eley (26), Alice M Gregory (27), Mingguang He (28) (29), Xiaohu Ding (28), Morten Bjerregaard-Andersen (30) (31) (32), Henning Beck-Nielsen (32), Morten Sodemann (33), Adam D Tarnoki (34) (35), David L Tarnoki (34) (35), Ariel Knafo-Noam (36), David Mankuta (37), Lior Abramson (36), S Alexandra Burt (38), Kelly L Klump (38), Judy L Silberg (39), Lindon J Eaves (39), Hermine H Maes (40), Robert F Krueger (41), Matt McGue (41), Shandell Pahlen (41), Margaret Gatz (42) (7), David A Butler (43), Meike Bartels (44), Toos CEM van Beijsterveldt (44), Jeffrey M Craig (45) (46), Richard Saffery (45) (46), Lise Dubois (47), Michel Boivin (48) (49), Mara Brendgen (50), Ginette Dionne (48), Frank Vitaro (51), Nicholas G Martin (52), Sarah E Medland (52), Grant W Montgomery (53), Gary E Swan (54), Ruth Krasnow (55), Per Tynelius (56), Paul Lichtenstein (7), Claire MA Haworth (57), Robert Plomin (26), Gombojav Bayasgalan (58), Danshiitsoodol Narandalai (59) (58), K Paige Harden (60), Elliot M Tucker-Drob (60), Timothy Spector (61), Massimo Mangino (61), Genevieve Lachance (61), Laura A Baker (42), Catherine Tuvblad (42) (62), Glen E Duncan (63), Dedra Buchwald (64), Gonneke Willemsen (44), Axel Skytthe (5), Kirsten O Kyvik (65) (66), Kaare Christensen (5) (67), Sevgi Y Öncel (68), Fazil Aliev (69), Finn Rasmussen (56), Jack H Goldberg (70), Thorkild IA Sørensen (71) (72), Dorret I Boomsma (44), Jaakko Kaprio (9) (73) (74), Karri Silventoinen (1) (6)

† deceased

Supplementary Table S1. Univariate analysis results for the proportion of height variance explained by additive genetic, common environmental and unique environmental factors by age, sex and geographic-cultural region.

	Boys						Girls					
	Additive genetics		Common environment		Unique environment		Additive genetics		Common environment		Unique environment	
	a^2	CI	c^2	CI	e^2	CI	a^2	CI	c^2	CI	e^2	CI
All cohorts												
Age 1	0.40	0.36-0.44	0.48	0.44-0.52	0.12	0.11-0.13	0.38	0.34-0.42	0.49	0.46-0.53	0.12	0.12-0.13
Age 2	0.53	0.48-0.57	0.38	0.33-0.42	0.10	0.09-0.10	0.54	0.50-0.59	0.37	0.32-0.41	0.09	0.08-0.10
Age 3	0.64	0.60-0.69	0.27	0.22-0.31	0.09	0.09-0.10	0.60	0.56-0.65	0.30	0.26-0.34	0.10	0.09-0.10
Age 4	0.54	0.49-0.59	0.37	0.32-0.42	0.09	0.09-0.10	0.49	0.45-0.54	0.41	0.36-0.46	0.09	0.09-0.10
Age 5	0.61	0.56-0.66	0.34	0.28-0.39	0.05	0.05-0.06	0.61	0.55-0.67	0.32	0.26-0.38	0.07	0.06-0.07
Age 6	0.53	0.45-0.61	0.41	0.32-0.48	0.07	0.060.07-	0.64	0.55-0.75	0.29	0.19-0.39	0.06	0.05-0.07
Age 7	0.59	0.55-0.64	0.35	0.30-0.39	0.06	0.06-0.07	0.63	0.58-0.68	0.30	0.25-0.34	0.07	0.07-0.08
Age 8	0.65	0.59-0.72	0.28	0.21-0.35	0.06	0.06-0.07	0.68	0.61-0.76	0.26	0.18-0.33	0.06	0.06-0.07
Age 9	0.70	0.63-0.77	0.23	0.16-0.30	0.07	0.06-0.07	0.64	0.58-0.71	0.30	0.23-0.36	0.06	0.06-0.07
Age 10	0.76	0.70-0.82	0.17	0.11-0.23	0.07	0.06-0.07	0.65	0.60-0.71	0.27	0.22-0.33	0.07	0.07-0.08
Age 11	0.74	0.67-0.80	0.18	0.11-0.24	0.09	0.08-0.09	0.77	0.70-0.84	0.17	0.10-0.23	0.07	0.06-0.07
Age 12	0.74	0.69-0.80	0.17	0.11-0.22	0.09	0.08-0.10	0.75	0.69-0.81	0.17	0.11-0.22	0.08	0.08-0.09
Age 13	0.72	0.64-0.80	0.21	0.12-0.29	0.07	0.07-0.08	0.73	0.65-0.82	0.17	0.08-0.26	0.10	0.09-0.11
Age 14	0.83	0.76-0.90	0.08	0.00-0.15	0.09	0.08-0.10	0.73	0.66-0.80	0.15	0.08-0.22	0.12	0.12-0.13
Age 15	0.68	0.60-0.76	0.23	0.15-0.30	0.10	0.09-0.11	0.76	0.68-0.85	0.14	0.04-0.22	0.10	0.09-0.11
Age 16	0.82	0.75-0.89	0.06	0.00-0.14	0.11	0.10-0.12	0.72	0.66-0.79	0.16	0.09-0.22	0.12	0.11-0.13
Age 17	0.77	0.71-0.84	0.13	0.06-0.19	0.10	0.10-0.11	0.68	0.61-0.75	0.19	0.12-0.25	0.14	0.13-0.15
Age 18	0.75	0.70-0.80	0.17	0.12-0.21	0.09	0.08-0.09	0.74	0.67-0.81	0.15	0.08-0.22	0.11	0.10-0.12
Age 19	0.76	0.70-0.82	0.14	0.08-0.20	0.10	0.09-0.11	0.73	0.66-0.80	0.15	0.07-0.21	0.13	0.12-0.14
Europe												
Age 1	0.42	0.38-0.47	0.46	0.42-0.50	0.12	0.11-0.12	0.40	0.36-0.45	0.47	0.43-0.51	0.12	0.12-0.13
Age 2	0.55	0.50-0.60	0.35	0.30-0.40	0.10	0.09-0.11	0.58	0.52-0.63	0.33	0.28-0.38	0.09	0.09-0.10
Age 3	0.65	0.60-0.70	0.26	0.21-0.30	0.10	0.09-0.10	0.59	0.55-0.64	0.30	0.26-0.35	0.10	0.10-0.11
Age 4	0.56	0.50-0.63	0.32	0.26-0.38	0.11	0.10-0.12	0.53	0.47-0.59	0.37	0.31-0.43	0.10	0.09-0.11
Age 5	0.63	0.57-0.70	0.32	0.25-0.38	0.05	0.05-0.06	0.63	0.56-0.70	0.31	0.23-0.37	0.06	0.06-0.07
Age 6	0.76	0.63-0.91	0.19	0.04-0.32	0.04	0.04-0.05	0.69	0.55-0.87	0.28	0.10-0.43	0.03	0.02-0.04
Age 7	0.61	0.56-0.66	0.32	0.27-0.37	0.06	0.06-0.07	0.64	0.60-0.70	0.28	0.23-0.33	0.07	0.07-0.08
Age 8	0.69	0.61-0.78	0.25	0.16-0.33	0.06	0.05-0.07	0.67	0.59-0.76	0.28	0.19-0.36	0.05	0.05-0.06
Age 9	0.72	0.63-0.81	0.23	0.13-0.31	0.05	0.05-0.06	0.62	0.54-0.70	0.33	0.24-0.40	0.06	0.05-0.07
Age 10	0.75	0.69-0.82	0.18	0.12-0.24	0.07	0.06-0.07	0.66	0.60-0.72	0.27	0.20-0.32	0.07	0.07-0.08

Age 11	0.79	0.71-0.85	0.13	0.05-0.21	0.08	0.07-0.09	0.75	0.68-0.83	0.18	0.10-0.25	0.07	0.07-0.08
Age 12	0.75	0.69-0.83	0.16	0.09-0.22	0.09	0.08-0.10	0.77	0.70-0.84	0.15	0.07-0.21	0.08	0.08-0.09
Age 13	0.72	0.63-0.82	0.22	0.12-0.31	0.06	0.05-0.07	0.75	0.66-0.86	0.17	0.07-0.27	0.07	0.06-0.08
Age 14	0.79	0.70-0.88	0.12	0.03-0.20	0.09	0.08-0.10	0.74	0.66-0.83	0.13	0.05-0.21	0.12	0.11-0.14
Age 15	0.66	0.57-0.76	0.24	0.14-0.33	0.10	0.09-0.11	0.70	0.60-0.80	0.20	0.10-0.30	0.10	0.09-0.11
Age 16	0.83	0.75-0.89	0.05	0.00-0.13	0.12	0.11-0.13	0.68	0.60-0.76	0.19	0.11-0.27	0.13	0.12-0.14
Age 17	0.82	0.74-0.90	0.08	0.00-0.16	0.10	0.09-0.11	0.70	0.62-0.78	0.15	0.08-0.23	0.15	0.14-0.16
Age 18	0.82	0.75-0.90	0.10	0.02-0.17	0.08	0.07-0.08	0.75	0.67-0.84	0.14	0.06-0.22	0.10	0.10-0.12
Age 19	0.86	0.77-0.91	0.05	0.00-0.14	0.10	0.09-0.11	0.78	0.70-0.87	0.11	0.02-0.19	0.11	0.10-0.12
North-America and Australia												
Age 1	0.28	0.06-0.34	0.52	0.26-0.70	0.21	0.16-0.28	0.50	0.25-0.82	0.30	0.00-0.53	0.20	0.16-0.27
Age 2	0.52	0.34-0.73	0.36	0.15-0.53	0.12	0.09-0.16	0.37	0.21-0.56	0.50	0.31-0.65	0.13	0.10-0.17
Age 3	0.50	0.38-0.65	0.42	0.27-0.54	0.08	0.06-0.10	0.47	0.34-0.63	0.41	0.25-0.53	0.12	0.10-0.15
Age 4	0.47	0.38-0.58	0.48	0.37-0.57	0.05	0.04-0.06	0.36	0.26-0.47	0.55	0.44-0.64	0.09	0.07-0.11
Age 5	0.61	0.49-0.76	0.34	0.19-0.47	0.05	0.04-0.06	0.50	0.37-0.66	0.43	0.26-0.55	0.08	0.06-0.10
Age 6	0.28	0.18-0.41	0.64	0.51-0.74	0.08	0.06-0.10	0.62	0.47-0.81	0.32	0.13-0.46	0.06	0.05-0.08
Age 7	0.47	0.35-0.64	0.48	0.32-0.61	0.04	0.03-0.06	0.55	0.41-0.72	0.39	0.22-0.53	0.06	0.04-0.08
Age 8	0.52	0.37-0.73	0.43	0.22-0.58	0.06	0.04-0.08	0.69	0.47-0.91	0.21	0.00-0.42	0.10	0.08-0.14
Age 9	0.59	0.46-0.75	0.31	0.16-0.44	0.09	0.08-0.12	0.62	0.48-0.79	0.31	0.14-0.45	0.07	0.06-0.09
Age 10	0.83	0.63-0.95	0.11	0.00-0.30	0.07	0.05-0.09	0.46	0.31-0.66	0.45	0.25-0.59	0.10	0.07-0.12
Age 11	0.47	0.32-0.65	0.40	0.22-0.54	0.13	0.10-0.17	0.72	0.56-0.93	0.23	0.02-0.40	0.04	0.04-0.06
Age 12	0.70	0.58-0.84	0.21	0.06-0.33	0.10	0.08-0.11	0.67	0.56-0.79	0.23	0.10-0.34	0.11	0.09-0.12
Age 13	0.71	0.54-0.90	0.17	0.00-0.34	0.11	0.09-0.14	0.70	0.52-0.87	0.16	0.00-0.33	0.15	0.12-0.18
Age 14	0.91	0.79-0.92	0.00	0.00-0.11	0.09	0.08-0.11	0.67	0.55-0.81	0.20	0.06-0.32	0.13	0.11-0.14
Age 15	0.75	0.60-0.91	0.16	0.00-0.31	0.09	0.08-0.11	0.88	0.72-0.91	0.01	0.00-0.17	0.11	0.09-0.13
Age 16	0.81	0.67-0.91	0.09	0.00-0.22	0.10	0.09-0.12	0.81	0.68-0.91	0.09	0.00-0.22	0.10	0.09-0.12
Age 17	0.69	0.59-0.80	0.21	0.10-0.31	0.10	0.09-0.11	0.59	0.46-0.75	0.31	0.15-0.44	0.10	0.09-0.12
Age 18	0.70	0.64-0.76	0.21	0.15-0.27	0.09	0.08-0.10	0.73	0.60-0.90	0.16	0.00-0.30	0.11	0.09-0.12
Age 19	0.66	0.57-0.75	0.24	0.15-0.32	0.10	0.09-0.12	0.66	0.53-0.82	0.19	0.03-0.32	0.15	0.13-0.17
East-Asia												
Age 1	0.30	0.21-0.41	0.58	0.47-0.67	0.12	0.11-0.14	0.33	0.24-0.44	0.56	0.45-0.65	0.11	0.10-0.12
Age 2	0.39	0.29-0.51	0.53	0.41-0.63	0.08	0.07-0.09	0.42	0.32-0.55	0.50	0.38-0.60	0.07	0.06-0.08
Age 3	0.66	0.52-0.82	0.26	0.09-0.39	0.09	0.08-0.10	0.70	0.57-0.86	0.23	0.07-0.36	0.07	0.06-0.08
Age 4	0.47	0.33-0.65	0.46	0.27-0.60	0.08	0.06-0.09	0.47	0.32-0.66	0.45	0.25-0.59	0.09	0.07-0.10
Age 5	0.33	0.22-0.49	0.58	0.42-0.70	0.09	0.07-0.11	0.72	0.52-0.93	0.20	0.00-0.40	0.08	0.07-0.10
Age 6	0.63	0.47-0.82	0.30	0.10-0.45	0.08	0.06-0.09	0.68	0.50-0.92	0.23	0.00-0.42	0.08	0.07-0.10
Age 7	0.58	0.44-0.76	0.35	0.17-0.49	0.07	0.06-0.08	0.61	0.45-0.82	0.30	0.09-0.46	0.08	0.07-0.10

Age 8	0.64	0.49-0.83	0.28	0.10-0.43	0.08	0.07-0.09	0.64	0.48-0.86	0.28	0.07-0.45	0.07	0.06-0.09
Age 9	0.79	0.62-0.92	0.12	0.00-0.30	0.09	0.07-0.10	0.77	0.59-0.94	0.16	0.00-0.34	0.07	0.06-0.08
Age 10	0.73	0.57-0.93	0.20	0.00-0.36	0.07	0.06-0.08	0.82	0.64-0.94	0.11	0.00-0.30	0.06	0.05-0.07
Age 11	0.69	0.53-0.89	0.23	0.03-0.39	0.09	0.08-0.10	0.85	0.67-0.94	0.08	0.00-0.27	0.07	0.06-0.08
Age 12	0.69	0.50-0.91	0.21	0.00-0.41	0.10	0.08-0.12	0.61	0.44-0.83	0.33	0.10-0.49	0.07	0.06-0.08
Age 13	0.57	0.31-0.96	0.38	0.00-0.64	0.05	0.03-0.07	0.60	0.27-0.93	0.31	0.00-0.64	0.09	0.06-0.13
Age 14	0.61	0.27-0.94	0.31	0.00-0.65	0.08	0.05-0.14	0.79	0.24-0.91	0.08	0.00-0.63	0.13	0.08-0.21
Age 15	0.25	0.10-0.56	0.71	0.39-0.86	0.04	0.03-0.08	0.72	0.34-0.94	0.18	0.00-0.57	0.09	0.06-0.15
Age 16	0.56	0.26-0.97	0.40	0.00-0.71	0.04	0.02-0.07	0.91	0.38-0.95	0.02	0.00-0.55	0.07	0.05-0.12
Age 17	0.38	0.09-0.94	0.54	0.00-0.82	0.08	0.04-0.15	0.32	0.00-0.88	0.50	0.00-0.84	0.18	0.11-0.29
Age 18	0.89	0.45-0.93	0.00	0.00-0.43	0.11	0.07-0.20	0.40	0.00-0.92	0.47	0.00-0.86	0.13	0.08-0.21
Age 19	0.23	0.01-0.94	0.70	0.00-0.91	0.07	0.04-0.12	0.82	0.27-0.92	0.05	0.00-0.58	0.13	0.08-0.23

CI: 95% confidence intervals

Supplementary Table S2. Model fit statistics for height from 1 to 19 years of age (all cohorts together).

	Saturated model		Full ACE sex-limitation		Same parameters for boys and girls		Scale model for sex		No sex-specific genetic effects		AE model		Scale model for region	
	-2LL	d.f.	Δ-2LL[1]	p-value[1]	Δ-2LL[2]	p-value[2]	Δ-2LL[3]	p-value[3]	Δ-2LL[4]	p-value[4]	Δ-2LL[5]	p-value[5]	Δ-2LL[6]	p-value[6]
Age 1	143430	31007	24.4	0.0410	0.5	0.9189	0.5	0.4795	9.6	0.0019	677.7	<0.0001	37.7	<0.0001
Age 2	122000	24865	21.9	0.0807	1.9	0.5934	1.9	0.1681	0.6	0.4386	325.2	<0.0001	38.5	<0.0001
Age 3	177673	34031	36.8	0.0008	6.5	0.0897	4.6	0.0320	1.9	0.1681	244.4	<0.0001	40.9	<0.0001
Age 4	107889	19265	18.4	0.1892	4.4	0.2214	1.8	0.1797	9.3	0.0023	293.9	<0.0001	64.1	<0.0001
Age 5	89657	15779	18.9	0.1704	23.0	0.0000	11.4	0.0007	0.1	0.8065	169.4	<0.0001	43.9	<0.0001
Age 6	35088	5949	36.0	0.0010	6.9	0.0758	3.9	0.0486	1.0	0.3078	84.4	<0.0001	76.2	<0.0001
Age 7	159173	27107	24.5	0.0398	16.8	0.0008	16.0	0.0001	20.1	<0.0001	272.4	<0.0001	18.0	0.0012
Age 8	72530	12345	38.0	0.0005	2.6	0.4505	0.3	0.5598	6.3	0.0119	81.6	<0.0001	36.9	<0.0001
Age 9	81358	13487	20.2	0.1240	3.9	0.2680	3.9	0.0472	4.6	0.0320	83.4	<0.0001	40.9	<0.0001
Age 10	138344	22501	13.4	0.4953	8157	<0.0001	11.2	0.0008	5.3	0.0213	100.4	<0.0001	13.9	0.0076
Age 11	111027	17557	19.8	0.1366	47.1	<0.0001	31.9	<0.0001	10.2	0.0014	44.0	<0.0001	39.5	<0.0001
Age 12	150718	23447	17.1	0.2509	8.4	0.0384	2.5	0.1138	6.3	0.0121	52.4	<0.0001	23.0	0.0001
Age 13	61446	9319	33.7	0.0023	172.0	<0.0001	13.1	0.0003	5.1	0.0246	32.2	<0.0001	67.5	<0.0001
Age 14	118323	18259	19.5	0.1467	752.3	<0.0001	37.5	<0.0001	3.6	0.0578	19.7	0.0001	4.6	0.3309
Age 15	67906	10581	16.9	0.2615	374.1	<0.0001	2.6	0.1035	8.2	0.0042	33.8	<0.0001	20.1	0.0005
Age 16	105515	16789	18.6	0.1808	202.2	<0.0001	4.4	0.0359	2.7	0.1003	20.8	<0.0001	31.2	<0.0001
Age 17	118205	19007	10.4	0.7324	123.8	<0.0001	34.7	<0.0001	5.1	0.0239	38.1	<0.0001	25.5	<0.0001
Age 18	137054	22401	36.2	0.0010	6457	<0.0001	23.6	<0.0001	15.2	0.0001	57.5	<0.0001	18.0	0.0012
Age 19	103952	16869	22.5	0.0689	73.9	<0.0001	18.7	<0.0001	9.0	0.0027	30.3	<0.0001	35.5	<0.0001

The fit of nested models was compared by calculating differences in -2 log-likelihood values (Δ-2LL), which follows the χ^2-distribution with a difference in degrees of freedom (Δd.f.) that corresponds to the difference in the number of free parameters estimated.

[1]Compared to saturated model (Δd.f.=14); [2]Compared to the full ACE sex-limitation model (Δd.f.=3); [3]Compared to the full ACE sex-limitation model (Δd.f.=1); [4]Compared to the full ACE sex-limitation model (Δd.f.=1); [5]Compared to the full ACE sex-limitation model (Δd.f.=2)[6]Compared to the model with free parameter estimates in all regions (Δd.f.=4).

Supplementary Table S3. Parameter estimates and age modification effects with 95% confidence intervals of height by geographic-cultural region in boys and girls.[1]

	Additive genetics		Common environment		Specific environment	
	a	95% CI	c	95% CI	e	95% CI
All boys						
Estimates at 9 years of age	5.73	5.65, 5.81	3.05	2.90, 3.20	1.82	1.80, 1.84
Linear age effects per 10 years	2.73	2.66, 2.80	0.39	0.54, 0.53	0.76	0.74, 0.78
Quadratic age effects per 10 years	-0.26	-0.27, -0.24	-0.09	-0.12, -0.07	-0.03	-0.03, -0.02
All girls						
Estimates at 9 years of age	5.51	5.42, 5.59	3.11	2.96, 3.26	1.80	1.78, 1.82
Linear age effects per 10 years	2.24	2.16, 2.32	0.33	0.16, 0.48	0.72	0.70, 0.74
Quadratic age effects per 10 years	-0.28	-0.30, -0.26	-0.11	-0.15, -0.08	-0.03	-0.03, -0.02
European boys						
Estimates at 9 years of age	5.73	5.63, 5.82	2.83	2.64, 3.02	1.78	1.75, 1.80
Linear age effects per 10 years	2.82	2.73, 2.91	0.28	0.08, 0.47	0.78	0.75, 0.81
Quadratic age effects per 10 years	-0.24	-0.26, -0.23	-0.11	-0.15, -0.08	-0.02	-0.03, -0.01
European girls						
Estimates at 9 years of age	5.49	5.40, 5.59	3.12	2.93, 3.29	1.79	1.77, 1.82
Linear age effects per 10 years	2.20	2.11, 2.29	0.36	0.18, 0.53	0.69	0.67, 0.72
Quadratic age effects per 10 years	-0.29	-0.31, -0.27	-0.13	-0.17, -0.09	-0.03	-0.04, -0.03
North-American and Australian boys						
Estimates at 9 years of age	6.27	5.65, 6.53	4.64	4.19, 6.78	2.31	2.25, 2.38
Linear age effects per 10 years	2.79	2.59, 4.35	0.69	-3.14, 1.00	0.77	0.72, 0.83
Quadratic age effects per 10 years	-0.35	-0.40, -0.20	-0.23	-0.32, -0.11	-0.10	-0.11, -0.09
North-American and Australian girls						
Estimates at 9 years of age	5.99	5.77, 6.22	4.55	4.16, 4.92	2.20	2.14, 2.27
Linear age effects per 10 years	2.42	2.18, 2.66	-2.13	-2.53, -1.66	0.66	0.59, 0.74
Quadratic age effects per 10 years	-0.33	-0.38, -0.27	-0.67	-0.74, -0.61	-0.05	-0.07, -0.04
East-Asian boys						
Estimates at 9 years of age	4.62	4.40, 4.84	3.00	2.63, 3.32	1.56	1.52, 1.60
Linear age effects per 10 years	2.25	1.76, 2.75	0.79	0.01, 1.49	0.70	0.57, 0.84
Quadratic age effects per 10 years	-0.13	-0.20, -0.06	0.01	-0.08, 0.10	0.02	0.00, 0.04
East-Asian girls						
Estimates at 9 years of age	5.03	4.77, 5.31	2.17	1.41, 2.69	1.50	1.46, 1.53
Linear age effects per 10 years	2.09	1.70, 2.47	-0.11	-0.99, 0.59	0.64	0.54, 0.74
Quadratic age effects per 10 years	-0.22	-0.28, -0.16	0.04	-0.05, 0.13	0.01	0.00, 0.03

[1]Age is centered as having zero at 9 years of age. Thus ages before 9 are marked as numbers -1 to -8 for linear effects and 1 to 64 for quadratic effects and after 9 years as 1 to 10 and 1 to 100, respectively.

Supplementary Table S4. Number of pairs and within-pair correlations of height from 1 to 19 years assessed by zygosity, age, sex and geographic-cultural region.

	Boys MZ		Boys DZ		Girls MZ		Girls DZ		Opposite-sex DZ	
	N	r	N	r	N	r	N	r	N	r
All cohorts										
Age 1	2821	0.88	2579	0.67	3013	0.88	2391	0.67	4712	0.65
Age 2	2282	0.91	2177	0.65	2328	0.91	1909	0.64	3749	0.63
Age 3	2872	0.91	2912	0.58	3229	0.89	2743	0.61	5272	0.59
Age 4	1636	0.90	1713	0.64	1677	0.90	1598	0.66	3021	0.62
Age 5	1369	0.94	1404	0.65	1441	0.93	1247	0.62	2441	0.64
Age 6	650	0.93	557	0.68	567	0.93	460	0.65	753	0.63
Age 7	2438	0.93	2246	0.64	2671	0.92	2183	0.63	4028	0.58
Age 8	1219	0.93	1033	0.62	1250	0.93	889	0.61	1794	0.57
Age 9	1366	0.93	1099	0.59	1347	0.94	1004	0.62	1940	0.57
Age 10	2118	0.93	1836	0.56	2262	0.93	1727	0.60	3320	0.53
Age 11	1731	0.91	1468	0.55	1840	0.93	1311	0.57	2441	0.50
Age 12	2351	0.91	1868	0.55	2476	0.93	1880	0.57	3161	0.50
Age 13	920	0.93	800	0.59	895	0.89	752	0.57	1305	0.47
Age 14	1656	0.91	1471	0.48	1865	0.88	1508	0.51	2642	0.38
Age 15	1022	0.90	1002	0.56	1070	0.90	950	0.51	1259	0.40
Age 16	1435	0.89	1416	0.49	1770	0.88	1475	0.53	2311	0.39
Age 17	1925	0.90	1842	0.50	1903	0.86	1551	0.52	2295	0.47
Age 18	3325	0.91	3228	0.53	1681	0.89	1363	0.52	1616	0.43
Age 19	1855	0.90	1804	0.51	1828	0.88	1405	0.51	1555	0.44
Europe										
Age 1	2086	0.89	2209	0.67	2185	0.88	2022	0.66	4084	0.66
Age 2	1705	0.90	1825	0.63	1717	0.91	1577	0.62	3297	0.62
Age 3	2135	0.91	2454	0.58	2389	0.89	2294	0.60	4583	0.58
Age 4	1158	0.89	1307	0.60	1188	0.90	1227	0.63	2314	0.59
Age 5	945	0.95	1094	0.62	1009	0.94	965	0.61	1936	0.61
Age 6	230	0.95	275	0.60	97	0.97	187	0.60	318	0.56
Age 7	1902	0.94	1910	0.62	2067	0.93	1861	0.61	3512	0.56
Age 8	721	0.94	774	0.60	677	0.95	641	0.63	1343	0.55
Age 9	752	0.94	717	0.57	649	0.94	648	0.63	1274	0.57
Age 10	1549	0.93	1509	0.55	1614	0.93	1434	0.59	2786	0.53
Age 11	1098	0.92	1153	0.53	1132	0.93	1028	0.56	1968	0.50
Age 12	1492	0.91	1399	0.53	1567	0.92	1341	0.53	2560	0.51
Age 13	617	0.94	579	0.60	538	0.92	536	0.59	930	0.48
Age 14	1142	0.91	1059	0.48	1287	0.88	1082	0.50	1906	0.39
Age 15	675	0.91	744	0.55	737	0.90	659	0.54	944	0.40
Age 16	999	0.88	1033	0.47	1286	0.87	1101	0.53	1742	0.39
Age 17	1189	0.90	1217	0.49	1505	0.85	1265	0.51	1983	0.45
Age 18	1385	0.92	1275	0.50	1120	0.90	1035	0.52	1194	0.45
Age 19	875	0.91	979	0.47	1184	0.90	1050	0.51	1114	0.44
North-America and Australia										

Age 1	104	0.81	95	0.63	125	0.82	92	0.52	149	0.52
Age 2	133	0.86	134	0.62	136	0.87	117	0.70	73	0.62
Age 3	171	0.90	221	0.66	204	0.87	206	0.66	266	0.69
Age 4	209	0.94	281	0.73	211	0.91	244	0.73	501	0.67
Age 5	176	0.94	194	0.69	162	0.92	173	0.69	340	0.76
Age 6	123	0.93	142	0.77	121	0.93	138	0.68	267	0.70
Age 7	107	0.95	130	0.74	104	0.92	139	0.72	233	0.74
Age 8	101	0.93	107	0.66	98	0.90	111	0.55	263	0.66
Age 9	216	0.90	242	0.62	226	0.93	214	0.58	482	0.61
Age 10	134	0.93	144	0.52	144	0.89	123	0.66	283	0.61
Age 11	182	0.83	158	0.65	196	0.94	145	0.61	271	0.47
Age 12	510	0.91	362	0.55	508	0.88	429	0.56	453	0.51
Age 13	211	0.90	193	0.54	259	0.83	191	0.52	316	0.45
Age 14	466	0.91	393	0.45	526	0.88	411	0.52	704	0.37
Age 15	310	0.89	239	0.54	278	0.89	272	0.46	279	0.39
Age 16	398	0.89	366	0.51	432	0.90	361	0.51	542	0.41
Age 17	706	0.89	607	0.53	350	0.90	271	0.56	292	0.54
Age 18	1897	0.91	1939	0.55	510	0.90	313	0.51	391	0.41
Age 19	939	0.90	815	0.56	602	0.86	339	0.51	415	0.42
East-Asia										
Age 1	625	0.88	265	0.71	695	0.90	266	0.71	433	0.72
Age 2	438	0.92	208	0.75	466	0.92	206	0.73	337	0.71
Age 3	561	0.91	232	0.60	630	0.92	238	0.61	401	0.59
Age 4	268	0.92	123	0.67	274	0.91	124	0.68	204	0.71
Age 5	244	0.91	112	0.78	267	0.92	105	0.59	158	0.48
Age 6	289	0.91	128	0.67	340	0.92	119	0.59	152	0.47
Age 7	395	0.93	139	0.69	464	0.92	129	0.64	172	0.40
Age 8	394	0.91	142	0.67	475	0.93	134	0.62	171	0.45
Age 9	396	0.90	133	0.60	472	0.93	131	0.59	169	0.40
Age 10	419	0.93	158	0.61	489	0.93	143	0.56	204	0.40
Age 11	451	0.91	156	0.61	511	0.93	138	0.54	201	0.48
Age 12	349	0.90	107	0.59	401	0.93	110	0.66	148	0.41
Age 13	92	0.94	28	0.69	98	0.89	25	0.57	59	0.52
Age 14	48	0.90	19	0.69	52	0.87	15	0.45	32	0.15
Age 15	35	0.95	18	0.91	53	0.86	18	0.65	31	0.49
Age 16	36	0.95	13	0.83	52	0.93	12	0.50	22	-0.16
Age 17	29	0.92	15	0.71	44	0.84	11	0.66	12	0.51
Age 18	37	0.88	10	0.47	45	0.87	8	0.77	24	0.13
Age 19	38	0.92	9	0.77	38	0.88	14	0.48	23	0.50
